# Agreement Between Artificial Intelligence-Based and Expert Consensus Implant Planning Using Cone-Beam Computed Tomography: A Retrospective Observational Study

**DOI:** 10.7759/cureus.117591

**Published:** 2026-10-07

**Authors:** Rahul Das, Satyavolu S Siva, Sunitha Kaggal, Jignesh Tate, Jay Gohil, Rajesh K Gupta, Ifath Nazia Ghori

**Affiliations:** 1 Department of Dentistry, PKG Medical College and Hospital, Kolkata, IND; 2 Department of Periodontics, Drs. Sudha and Nageswara Rao Siddhartha Institute of Dental Sciences, Gannavaram, IND; 3 Department of Orthodontics and Dentofacial Orthopedics, Sri Hasanamba Dental College and Hospital, Hassan, IND; 4 Department of Periodontics, Bharati Vidyapeeth (Deemed to be University) Dental College and Hospital, Navi Mumbai, IND; 5 Department of Prosthodontics, K. M. Shah Dental College and Hospital, Sumandeep Vidyapeeth (Deemed to be University), Vadodara, IND; 6 Department of Periodontics, Maharishi Markendeshwar College of Dental Sciences and Research, Maharshi Markandeshwar (Deemed to be University), Mullana, IND; 7 Department of Computer Science, Jazan University, Jazan, SAU

**Keywords:** artificial intelligence, cone-beam computed tomography, dental implants, retrospective studies, treatment planning

## Abstract

Introduction: Artificial intelligence (AI) is increasingly being incorporated into digital implant dentistry, but the quantitative validation of AI-generated implant plans against expert consensus remains limited. Therefore, this study aimed to evaluate the agreement between an AI-based implant planning system and an expert consensus plan using cone-beam computed tomography (CBCT) images of posterior edentulous sites.

Materials and methods: This cross-sectional, observational study included 50 posterior edentulous sites from 50 adult patients. Two experienced implantologists independently planned implant length, diameter, mesiodistal position, and angulation, and a consensus plan was established as the reference standard. The same datasets were analyzed using an AI-based implant planning system. Agreement was assessed using the intraclass correlation coefficient (ICC), mean absolute error (MAE), Bland-Altman analysis, Cohen’s kappa, and predefined clinical tolerance limits.

Results: Categorical agreement was 86% for implant length, 90% for diameter, 84% for position, and 88% for angulation. The ICC values were 0.91 (95% CI, 0.85-0.95) for length, 0.87 (0.78-0.92) for diameter, 0.85 (0.75-0.91) for position, and 0.80 (0.68-0.88) for angulation. The mean differences between AI and consensus were 0.1 mm for length, 0.1 mm for diameter, −0.1 mm for position, and 0.7^o^ for angulation; none were statistically significant (all p > 0.05). Clinically acceptable agreement was observed in 94%, 90%, 88%, and 82% of cases, respectively.

Conclusion: AI-based implant planning demonstrated good-to-excellent agreement with expert consensus planning across implant dimensions and three-dimensional positioning parameters. The findings indicate substantial concordance between AI-generated and expert-derived planning recommendations under the conditions evaluated. However, agreement with expert planning should not be interpreted as evidence of clinical or surgical accuracy. Prospective validation against actual postoperative implant positions is required to determine clinical accuracy.

## Introduction

Dental implant therapy is an established treatment option for the rehabilitation of edentulous sites, and predictable outcomes depend largely on accurate preoperative assessment and three-dimensional treatment planning [1]. The appropriate selection of implant dimensions and precise determination of implant position and angulation are essential for achieving adequate primary stability, maintaining appropriate distances from adjacent anatomical structures, and ensuring a prosthetically favorable implant placement [2]. Cone-beam computed tomography (CBCT) has become an important imaging modality in implant planning because it provides three-dimensional (3D) visualization of the available alveolar bone and surrounding anatomical structures, thereby facilitating a more comprehensive assessment than conventional two-dimensional radiography [3].

Despite advances in digital implant planning, conventional treatment planning remains dependent on clinicians’ interpretation of radiographic information and clinical experience. Manual identification of anatomical landmarks and determination of implant dimensions and spatial orientation may be influenced by operator expertise, resulting in variability between clinicians. These limitations have encouraged the development of computer-assisted and automated approaches that can improve standardization and potentially reduce the time required for treatment planning [3,4].

Artificial intelligence (AI), particularly deep learning-based technology, has emerged as a promising tool in the field of digital dentistry [5]. AI systems can process CBCT datasets, identify and segment relevant anatomical structures, and assist in generating implant-planning recommendations [6]. In a recent retrospective CBCT-based comparative study, Jadhav et al. reported lower angular and linear deviations and substantially reduced planning time with AI-assisted planning compared with manual clinician-based assessment, supporting the potential role of AI as a clinical decision support tool [7]. However, the authors emphasized the need for stronger evidence and appropriate clinical validation before widespread adoption. Elgarba et al. recently demonstrated that an AI-driven system could generate clinically acceptable virtual implant plans comparable to human expert planning while requiring substantially less planning time and demonstrating greater consistency [8]. However, further quantitative validation using robust agreement methods and clinically defined tolerance limits is necessary before AI-generated implant plans can be considered interchangeable with expert-derived treatment plans.

The agreement between AI-based recommendations and expert planning is particularly important because statistical association alone does not demonstrate that the two planning approaches provide clinically interchangeable results [9]. Therefore, an assessment of both the magnitude and distribution of the differences is necessary. Measures such as the intraclass correlation coefficient, mean absolute error, and Bland-Altman analysis can provide complementary information regarding the agreement between continuous planning parameters, whereas categorical agreement can determine the consistency of recommendations within clinically relevant groups [10].

Therefore, this study aimed to evaluate the agreement between an AI-based implant planning system and an expert consensus plan using CBCT images of posterior edentulous sites. The primary objective of this study was to evaluate the agreement between AI-generated implant planning and an expert consensus planning reference standard using CBCT images of posterior edentulous sites. Specifically, agreement in implant length, diameter, mesiodistal position, and angulation was assessed using continuous, categorical, and predefined clinically acceptable tolerance measures. Clinical accuracy relative to the actual surgically placed implant was not evaluated.

## Materials and methods

Study design and study setting

This retrospective, cross-sectional, observational study was conducted at the Department of Periodontics, Bharati Vidyapeeth (Deemed to be University) Dental College and Hospital, Navi Mumbai, Maharashtra, India, over a period of one year between October 2021 and October 2022. The study protocol was approved by the Institutional Ethics Committee of the College (Reference number: BVDUDC&H-IEC S134/2021 dated September 18, 2021). Since the investigation involved a retrospective evaluation of previously acquired CBCT records and did not involve any additional radiographic exposure or clinical intervention, the requirement for individual informed consent was waived in accordance with institutional ethical requirements. All records were anonymized before analysis, and no personally identifiable patient information was included in the study database.

Study population

Eligibility Criteria

Patients aged ≥ 18 years with a single posterior edentulous site planned for implant-supported rehabilitation were considered eligible. The selected site was required to have a CBCT examination performed within three months before implant treatment planning. Only datasets with adequate image quality and complete visualization of the edentulous ridge and relevant surrounding anatomical structures were included in the study. Records were excluded if the selected site had a history of previous implant placement or bone grafting. Patients with metabolic bone disease or a history of head and neck radiotherapy were also excluded from the study. CBCT datasets with severe metal or motion artifacts, as well as those with incomplete visualization of clinically relevant anatomical structures, including the inferior alveolar nerve or maxillary sinus, were excluded from the analysis.

Sample Size

Sample size was determined using a precision-based approach rather than a conventional hypothesis-testing framework, in accordance with recommended methods for agreement and reliability studies. For the continuous outcomes assessed using intraclass correlation coefficients (ICC), an anticipated ICC of 0.80, two raters (AI versus consensus assessment), and a target 95% confidence interval (CI) half-width of ≤0.15 were assumed. For an ICC estimated from two raters with a prespecified confidence-interval half-width, the required sample size was approximated using the following formula: \[ n = 1 + \frac{4z_{1-\alpha/2}^{2}(1-\rho)^{2}\left[1+(k-1)\rho\right]^{2}} {k(k-1)h^{2}} \] where: n = required number of subjects/sites; z _1-α _= critical value from the standard normal distribution (1.96 for α = 0.05); ρ = anticipated intraclass correlation coefficient (ICC); k = number of raters or methods compared; h = desired 95% confidence interval half-width.

Using these assumptions, the calculated minimum sample size was approximately 46 sites. To provide a small margin for potential exclusions or unusable records, the final sample size was set at 50 edentulous sites. Thus, 50 adult patients with a single posterior edentulous site were included, with one edentulous site selected from each patient. The records were obtained from the institutional database during the predefined one-year study period. Each patient contributed only one edentulous site to avoid multiple observations from the same individual and to maintain the independence of the study units.

CBCT acquisition protocol

All CBCT examinations were obtained using a Carestream CBCT system (Carestream Dental LLC, Atlanta, Georgia, United States) according to a standardized imaging protocol. The scans were acquired with a field of view of 8 × 8 cm, voxel sizes ranging from 0.2 to 0.3 mm, tube voltage ranging from 90 to 120 kVp, and tube current ranging from 3 to 8 mA. The acquired images were exported in the Digital Imaging and Communications in Medicine (DICOM) format and subsequently imported into the implant planning software for expert and AI-based assessments. CBCT examinations were reviewed before inclusion to ensure adequate visualization of the edentulous ridge and surrounding anatomical structures required for implant planning. As this was a retrospective investigation, no additional CBCT examination was performed specifically for the study.

Expert assessment and reference standard

Two experienced implantologists, each with at least five years of clinical experience in implant dentistry (RD and RKG), independently reviewed all eligible CBCT datasets and, while blinded to each other’s assessments and implant-planning decisions, determined the optimal implant dimensions and three-dimensional position for each edentulous site. Implant length was categorized according to the available implant dimensions (8.0, 10.0, 11.5, and 13.0 mm), with each length treated as a distinct category for categorical agreement analysis. Implant position was planned based on the three-dimensional morphology and available bone, the relationship to relevant anatomical structures, and a prosthetically appropriate trajectory that provided adequate clearance from adjacent structures.

For categorical assessment of mesiodistal position, an implant placed at the predetermined prosthetically appropriate position within the edentulous space was classified as ideal, whereas positions directed toward the mesial or distal adjacent tooth were classified as mesial or distal, respectively. Quantitatively, implant position was recorded as the mesiodistal distance from a predefined fixed anatomical reference point, and implant angulation was measured as the angle between the long axis of the planned implant and the planned prosthetic or occlusal plane. After completing their independent assessments, the two examiners compared their findings and resolved disagreements through discussion and mutual agreement.

A single consensus plan was established for each edentulous site and used as the reference standard for comparison with the AI-generated implant plan. The consensus value represented the agreed assessment and was not calculated by averaging the two measurements; no third examiner was involved. For tolerance-based agreement analysis, exact agreement was required for implant diameter, whereas a difference of ≤1 mm was considered acceptable for implant length. These criteria were prespecified for the agreement analysis to reflect the categorical nature of the available implant diameter options and the continuous nature of implant length; the tolerance thresholds were intended for analytical classification and should not be interpreted as universally established clinical thresholds.

Inter-examiner reliability

Two implantologists initially performed their assessments independently to evaluate inter-examiner reliability. Inter-examiner reliability was assessed using the ICC for continuous planning parameters and Cohen’s kappa coefficient for categorical parameters. Inter-examiner reliability between the two implantologists was assessed before establishment of the consensus reference standard. The ICCs for implant length, diameter, position, and angulation were 0.94, 0.92, 0.91, and 0.89, respectively, indicating excellent agreement between the two examiners. The corresponding Cohen’s kappa coefficients for the categorical classifications were 0.90, 0.93, 0.88, and 0.89, respectively, demonstrating substantial to near-perfect agreement. For the ICC, the observed coefficients were interpreted using commonly applied reliability thresholds, with values of 0.75-0.90 considered indicative of good reliability and values >0.90 considered excellent reliability.

AI-based implant planning

The DICOM datasets used for the expert assessment were subsequently processed using Planmeca Romexis dental software (Planmeca Oy, Helsinki, Finland), incorporating its deep learning-based AI implant planning functionality. The Romexis platform provides integrated dental imaging and treatment-planning functions, including 3D imaging and implant planning. The AI system automatically analyzed the CBCT dataset, segmented the edentulous ridge and relevant adjacent anatomical structures, and generated recommendations for implant length, implant diameter, implant position, and implant angulation. The AI-generated recommendations were obtained without manual adjustment or modification by investigators. The resulting implant planning parameters were recorded and converted, where necessary, to the same measurement coordinate system used for the expert consensus plan to allow a direct comparison between the two planning approaches. The use of the same DICOM datasets for both approaches ensured that the AI and expert assessments were based on identical radiographic data.

Outcome measures

The primary outcomes were continuous measurements of the implant length, diameter, position, and angulation. The implant length and diameter were recorded in millimeters. For the secondary categorical analysis, implant length was classified as ≤8 mm, 10 mm, or ≥12 mm. The implant diameter was classified as narrow (3.3 mm), regular (3.75 or 4.3 mm), or wide (5.0 mm). The implant position was classified as ideal, mesial, or distal relative to the consensus reference position. Implant angulation was classified as <10^o^, 10^o^-20^o^, or >20^o^ [11,12].

Clinically acceptable agreement

Predefined, literature-informed tolerance limits were used to determine whether the differences between the AI-generated and expert consensus recommendations fell within the prespecified acceptable range for agreement. Implant length was considered within the predefined tolerance when the AI recommendation differed from the consensus value by no more than ±1 mm. Implant diameter required an exact match because it was assessed according to the discrete implant diameter categories available in the planning system. Implant position was considered within the predefined tolerance when the deviation from the consensus position was ≤1 mm, whereas implant angulation was considered within the predefined tolerance when the deviation was ≤5° [8,13]. These thresholds were selected based on previously reported linear and angular deviations in computer-assisted implant planning and placement and were used as analytical agreement criteria rather than as universally established definitions of clinical acceptability. The percentage of cases falling within these predefined tolerance limits was calculated for each planning parameter. Exact agreement was assessed separately to distinguish complete agreement from recommendations that differed numerically but remained within the predefined tolerance range.

Assessment of agreement

Agreement between the AI-generated recommendations and the expert consensus plans was assessed using complementary statistical approaches. For continuous planning parameters, the ICC was calculated using a two-way mixed-effects model for absolute agreement with a 95% CI. The mean absolute error (MAE) and standard deviation (SD) of the differences were calculated for each continuous planning parameter. Bland-Altman analysis was performed to evaluate systematic differences between the AI-generated and expert consensus measurements. The mean difference between the AI-generated and consensus measurements represented the bias, and the 95% limits of agreement (LoA) were calculated as the mean difference ±1.96 × SD of the differences. Regression analysis was performed to assess the presence of proportional bias. The ±1-mm tolerance was not intended to require the entire Bland-Altman LoA to fall within ±1 mm; rather, it was used to quantify the proportion of individual recommendations that remained within the prespecified tolerance.

Statistical analysis

The dataset was coded and anonymized before statistical analysis. The statistical analyses were performed by an independent statistician (SK), who was blinded to the identity of the patients and the corresponding planning methods during data analysis. The statistician was not involved in patient selection, CBCT assessment, expert planning, AI-based implant planning, or the establishment of the expert consensus reference standard. Statistical analyses were performed using IBM SPSS Statistics for Windows, version 28.0 (IBM Corp., Armonk, New York, United States). Continuous variables are summarized as mean ± SD. Normality of the paired differences was assessed using the Shapiro-Wilk test. The paired differences for implant length, implant diameter, implant position, and implant angulation were approximately normally distributed based on the Shapiro-Wilk assessment. Accordingly, paired t-tests were used to compare the AI-generated and expert consensus measurements. Paired t-tests were used when the paired differences were approximately normally distributed. A two-sided p < 0.05 was considered statistically significant. Effect size was expressed using Cohen’s dz (0.2 small, 0.5 medium, and 0.8 large). For categorical outcomes, percentage agreement with Wilson 95% CI and kappa coefficients were calculated; unweighted kappa was used for categorical classifications. The proportions of cases satisfying the predefined clinical tolerance criteria were calculated for implant length, diameter, position, and angulation.

Data management and quality control

All eligible CBCT datasets were assigned unique study identification numbers, and patient-identifying information was removed from the analytical dataset. Expert measurements, consensus values, AI-generated measurements, categorical classifications, and statistical results were structured using Microsoft Excel (Microsoft Corporation, Redmond, Washington, United States). The same measurement definitions, units, classification criteria, and clinical tolerance limits were consistently applied across all 50 edentulous sites.

## Results

A total of 50 edentulous sites from 50 patients were included. The age of the patients ranged from 42 to 60 years, with a mean age of 26.45 ± 5.65 years. The study population therefore represented middle-aged and older adults undergoing evaluation for posterior implant-supported rehabilitation. The reasons for tooth loss were not consistently documented in the retrospective database and were therefore not included as an analytical variable. Regarding sex distribution, 32 patients (64%) were male and 18 patients (36%) were female. The edentulous sites were located in the mandible in 22 cases (44%) and in the maxilla in 28 cases (56%). With respect to site type, 16 sites (32%) were premolar regions and 34 sites (68%) were molar regions (Table 1).

**Table 1:** Demographic details of the study samples (N=50) Data are presented as mean and standard deviation for age and frequency and percentage for categorical variables.

Characteristics		Value
Age, mean ± SD	Years	26.45 ± 5.65
Sex, n (%)	Male	32 (64%)
	Female	18 (36%)
Arch, n (%)	Mandible	22 ((44%)
	Maxilla	28 (56%)
Site, n (%)	Premolar	16 (32%)
	Molar	34 (68%)

Categorical agreement between AI and expert consensus

The highest percentage agreement was observed for implant diameter, followed by implant angulation, length, and position. Cohen’s kappa demonstrated substantial agreement for implant length (κ = 0.79, 95% CI: 0.61-0.97), implant position (κ = 0.75, 95% CI: 0.57-0.93), and implant angulation (κ = 0.78, 95% CI: 0.60-0.96), whereas agreement for implant diameter was almost perfect (κ = 0.82, 95% CI: 0.65-0.99). The z-test for kappa was statistically significant for all four parameters (all p < 0.001), indicating an agreement significantly greater than chance (Table 2).

**Table 2:** Categorical agreement between AI-generated and expert consensus implant recommendations (N = 50). Cohen’s kappa was tested using a z-test for agreement greater than zero; κ values were interpreted as <0.20, slight; 0.21–0.40, fair; 0.41–0.60, moderate; 0.61–0.80, substantial; and 0.81–1.00, almost perfect agreement. *p < 0.05 denotes statistical significance. AI: artificial intelligence; CI: confidence interval

Parameter	Category definition	% Agreement (95% CI)	Unweighted κ (95% CI)	z value	p-value	Kappa interpretation
Implant length	≤8, 10, ≥12 mm	86% (73.8–93.0)	0.79 (0.61–0.97)	6.12	< 0.001*	Substantial
Implant diameter	narrow, regular, wide	90% (78.6–95.7)	0.82 (0.65–0.99)	6.43	< 0.001*	Almost perfect
Implant position	ideal, mesial, distal	84% (71.5–91.7)	0.75 (0.57–0.93)	5.55	< 0.001*	Substantial
Implant angulation	<10^o^, 10–20^o^, >20^o^	88% (76.2–94.4)	0.78 (0.60–0.96)	5.79	< 0.001*	Substantial

Continuous agreement between AI and expert consensus

For implant length, the mean AI-recommended length was 10.4 ± 1.8 mm, compared with 10.3 ± 1.9 mm for the consensus plan. The ICC was 0.91 (95% CI: 0.85-0.95), indicating excellent agreement, and the MAE was 0.7 ± 0.5 mm. For implant diameter, the mean values were 4.2 ± 0.5 mm for the AI and 4.1 ± 0.6 mm for the consensus plan. The ICC was 0.87 (95% CI: 0.78-0.92), indicating good-to-excellent agreement, with an MAE of 0.3 ± 0.2 mm. For the implant position, the mean AI and consensus values were 5.2 ± 1.1 mm and 5.3 ± 1.2 mm, respectively. The ICC was 0.85 (95% CI: 0.75-0.91), indicating good agreement, with an MAE of 0.5 ± 0.4 mm. The corresponding means for implant angulation were 12.5 ± 5.6^o^ and 11.8 ± 6.0^o^, respectively. The ICC was 0.80 (95% CI: 0.68-0.88), indicating good agreement, with an MAE of 2.9^o^ ± 2.1^o^ (Table 3).

**Table 3:** Continuous agreement between AI-generated and expert consensus implant recommendations (N = 50). ICC was calculated using a two-way mixed-effects model for absolute agreement; p values correspond to the test of the ICC against zero; *p < 0.05 denotes statistical significance. AI: artificial intelligence, CI: confidence interval, ICC: intraclass correlation coefficient, MAE: mean absolute error

Parameter	AI, mean ± SD	Consensus, mean ± SD	ICC (95% CI)	F value (df)	p-value (ICC)	MAE, mean ± SD
Implant length (mm)	10.4 ± 1.8	10.3 ± 1.9	0.91 (0.85–0.95)	11.1 (49,49)	< 0.001*	0.7 ± 0.5
Implant diameter (mm)	4.2 ± 0.5	4.1 ± 0.6	0.87 (0.78–0.92)	7.7 (49.49)	< 0.001*	0.3 ± 0.2
Implant position (mm)	5.2 ± 1.1	5.3 ± 1.2	0.85 (0.75–0.91)	6.7 (49.49)	< 0.001 *	0.5 ± 0.4
Implant angulation (°)	12.5 ± 5.6	11.8 ± 6.0	0.80 (0.68–0.88)	5.0 (49.49)	< 0.001*	2.9 ± 2.1

Bland-Altman analysis and paired comparisons

Bland-Altman analysis demonstrated small mean differences between the AI-generated and expert consensus measurements for all four planning parameters. For implant length, the mean difference was 0.1 mm, with an SD of 0.9 mm and 95% limits of agreement (LoA) ranging from −1.7 to 1.9 mm. The paired comparison was not statistically significant (t = 0.79, p = 0.43), with a negligible effect size (Cohen’s d = 0.11). For implant diameter, the mean difference was 0.1 mm, with an SD of 0.4 mm and a 95% LoA ranging from −0.7 to 0.9 mm. No statistically significant difference was observed (t = 1.77, p = 0.08), and the effect size was small (Cohen’s d = 0.25). For implant position, the mean difference was −0.1 mm, with an SD of 0.6 mm and 95% LoA ranging from −1.3 to 1.1 mm. The paired comparison was not statistically significant (t = −1.18, p = 0.25), with a small effect size (Cohen’s d = −0.17). For implant angulation, the mean difference was 0.7^o^, with an SD of 3.8^o^, and the 95% LoA ranged from −6.7^o^ to 8.1^o^. The paired comparison was not statistically significant (t = 1.30, p = 0.20), with a small effect size (Cohen’s d = 0.18). Bland-Altman regression showed no significant proportional bias for any of the evaluated parameters (all p > 0.05) (Table 4, Figure 1).

**Table 4:** Bland–Altman analysis and paired comparisons between AI-generated and expert consensus measurements (N = 50). Paired t-tests were used to assess mean differences because the paired differences were treated as approximately normally distributed. Bland–Altman LoA were calculated as mean difference ± 1.96 × SD of differences. p < 0.05 was considered statistically significant. Bland–Altman regression showed no significant proportional bias for any parameter (all p > 0.05). AI: artificial intelligence, LoA: limits of agreement, MAE: mean absolute error, SD: standard deviation.

Parameter	Mean difference (AI − consensus)	SD of differences	95% LoA	t value (df = 49)	p-value	MAE ± SD	Cohen’s dz
Implant length (mm)	0.1	0.9	−1.7 to 1.9	0.79	0.43	0.7 ± 0.5	0.11
Implant diameter (mm)	0.1	0.4	−0.7 to 0.9	1.77	0.08	0.3 ± 0.2	0.25
Implant position (mm)	−0.1	0.6	−1.3 to 1.1	−1.18	0.25	0.5 ± 0.4	−0.17
Implant angulation (°)	0.7	3.8	−6.7 to 8.1	1.30	0.20	2.9 ± 2.1	0.18

**Figure 1:**
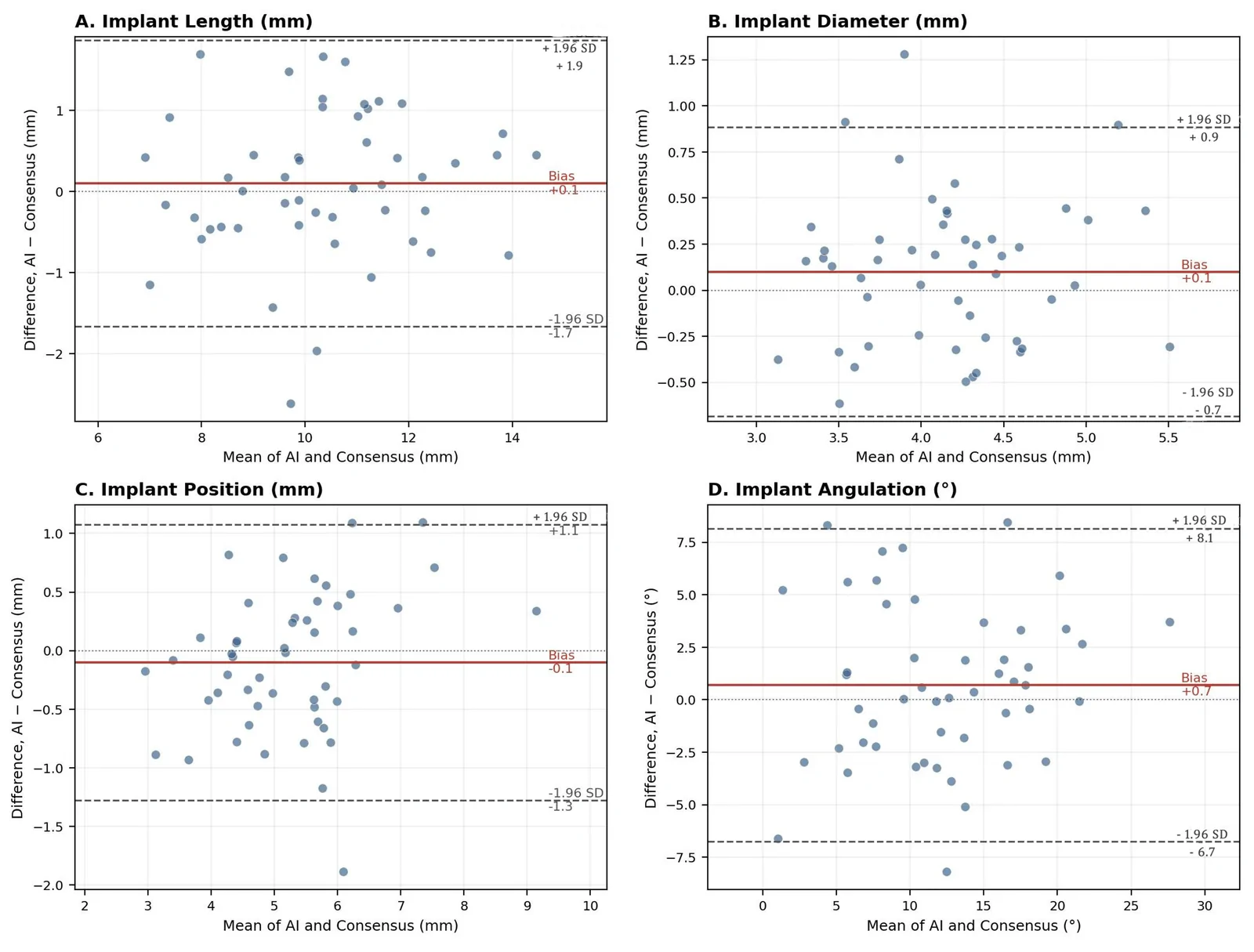
Bland–Altman plots comparing AI-generated and expert consensus implant planning measurements. Bland–Altman plots showing the agreement between AI-generated and expert consensus measurements for (A) implant length, (B) implant diameter, (C) implant position, and (D) implant angulation. The solid horizontal line represents the mean difference (bias), and the upper and lower dashed lines represent the 95% limits of agreement. No significant proportional bias was observed for any of the evaluated parameters (all p > 0.05). The plots were generated using IBM SPSS Statistics for Windows, version 28.0 (IBM Corp., Armonk, New York, United States).

Clinically acceptable tolerance agreement

For implant length, 47 (94%) cases were within the predefined ±1-mm tolerance, whereas exact agreement was observed in 36 (72%) cases. For implant diameter, 45 (90%) cases demonstrated exact agreement, which also represented clinically acceptable agreement because an exact diameter match was required. Regarding implant position, 44 (88%) cases were within the predefined ≤1-mm deviation from the consensus position, whereas exact agreement was observed in 31 (62%) cases. For implant angulation, 41 (82%) cases were within the predefined ≤5^o^ tolerance, whereas exact agreement was observed in 12 (24%) cases. These findings indicate a high proportion of clinically acceptable AI-generated recommendations, although the exact agreement was lower for implant position and particularly for implant angulation (Table 5).

**Table 5:** Clinically acceptable tolerance agreement between AI-generated and expert consensus implant recommendations (N = 50). Clinically acceptable agreement was defined a priori as implant length within ±1 mm, exact implant diameter match, implant position deviation ≤1 mm, and implant angulation deviation ≤5°. AI: artificial intelligence

Parameter	Tolerance definition	Within tolerance, n (%)	Exact agreement, n (%)
Implant length	±1 mm	47 (94%)	36 (72%)
Implant diameter	Exact match	45 (90%)	45 (90%)
Implant position	≤1 mm deviation	44 (88%)	31 (62%)
Implant angulation	≤5^o^ deviation	41 (82%)	12 (24%)

Overall findings

Overall, AI-generated implant planning demonstrated good-to-excellent agreement with the expert consensus plan across all evaluated implant planning parameters. The strongest continuous agreement was observed for implant length, followed by implant diameter and position, while implant angulation demonstrated good agreement. Categorical agreement was substantial to almost perfect across all parameters, and most AI-generated recommendations fell within the predefined clinically acceptable tolerance limits.

## Discussion

The present study demonstrated a high agreement between AI-generated implant planning and the expert consensus plan across implant length, diameter, position, and angulation. The categorical agreement ranged from 84% to 90%, with substantial agreement for implant length, position, and angulation, and almost perfect agreement for implant diameter. Continuous analysis similarly demonstrated good and excellent agreement. Importantly, the Bland-Altman analysis showed small mean differences for all parameters, and none of the paired comparisons demonstrated a statistically significant difference. These findings suggest that the AI-generated plans were closely aligned with consensus recommendations rather than merely demonstrating statistical correlations.

These findings are consistent with the rapidly expanding literature supporting AI as a potentially useful adjunct to digital implant planning [14]. Zaman et al., in a systematic review of 10 studies, reported high accuracy of AI systems for identifying teeth and edentulous regions on CBCT images, with reported accuracy ranging from 92% to 99.7% and precision and recall frequently exceeding 90% [15]. Their review also identified potential improvements in planning efficiency and reported higher implant success in one of the included studies compared with conventional planning. However, substantial heterogeneity in study designs, inconsistent outcome reporting, and limited ethical and clinical oversight have been highlighted as important barriers to widespread clinical implementation. These observations are consistent with the present findings, which demonstrated a high agreement between AI-generated and expert consensus implant plans, while also emphasizing the need for standardized quantitative assessment and clinically meaningful validation before AI-generated plans can be considered a substitute for expert clinical judgment.

Similarly, a scoping review identified 47 studies addressing AI in presurgical implant planning, but only eight studies specifically evaluated AI-based virtual implant placement. Importantly, the review found that none of the commercially available systems had demonstrated fully automated implant planning with adequate scientific validation at that time [5]. A particularly relevant comparison was provided by Elgarba et al., who evaluated an AI-driven virtual implant placement system against human intelligence using 50 CBCT and intraoral scan datasets involving single posterior mandibular tooth replacement [8]. Approximately 89% of AI-planned implants and 93% of human-planned implants were clinically acceptable. AI also selected implant dimensions close to those selected by experts and demonstrated substantially greater efficiency, requiring less than half the planning time of human operators. The present study extends these observations by focusing specifically on agreement with an expert consensus reference standard and quantifying agreement across four individual planning parameters. The excellent agreement observed for implant length and good-to-excellent agreement for diameter, position, and angulation support the feasibility of AI-assisted planning as a decision-support tool.

More recently, Jadhav et al. compared AI-assisted and manual implant treatment planning using CBCT data and reported lower angular and linear deviations with AI-assisted planning, along with substantially reduced planning time [7]. Their findings further support the potential of AI to improve the consistency and efficiency of implant treatment planning. However, direct comparisons between studies should be undertaken cautiously because the AI systems, reference standards, anatomical sites, outcome definitions, and statistical approaches differed. In particular, agreement with an expert plan is not equivalent to accuracy against surgically placed implants, and this distinction is important when interpreting the present findings.

Elgarba et al. reported that AI-generated implant plans were clinically acceptable in 95% of cases and required substantially less planning time than human expert planning (198 ± 33 vs. 435 ± 92 seconds), while also demonstrating greater consistency [16]. These findings were further supported by another recent study by Elgarba et al., in which no significant differences were found in implant location or dimension selection between AI-generated plans, expert plans, and clinically placed implants [17]. AI was also significantly faster and demonstrated excellent repeatability. The present findings are consistent with this evidence, demonstrating good-to-excellent agreement between AI-generated recommendations and expert consensus plans. However, the present study extends previous work by quantitatively assessing the agreement across implant length, diameter, position, and angulation using ICC, MAE, Bland-Altman analysis, categorical agreement, and predefined clinical tolerance limits.

Although the Bland-Altman 95% LoA extended beyond the predefined ±1-mm tolerance for linear parameters, this finding reflects the presence of individual cases with larger discrepancies and should be distinguished from the mean bias. The predefined tolerance analysis demonstrated the proportion of cases meeting the specified agreement criterion, whereas the Bland-Altman analysis characterized the overall distribution and variability of differences. Accordingly, the findings support overall agreement with some individual deviations, rather than implying that every AI recommendation was interchangeable with the expert consensus plan.

The relatively lower exact agreement for implant angulation warrants particular consideration. Angulation is inherently sensitive to small changes in the selected 3D implant trajectory and may vary according to the prosthetic orientation, available bone, and interpretation of surrounding anatomical structures. Although small angular differences may occur between planning approaches, implant angulation remains clinically relevant because it influences the restorative trajectory and the relationship of the implant with adjacent anatomical structures. Thus, the observed variability in exact angular recommendations highlights the need for careful clinical evaluation of AI-generated implant angulation rather than relying on the AI recommendation without expert assessment.

Elgarba et al. further demonstrated the clinical feasibility of AI-driven virtual implant planning in 32 posterior mandibular single-implant cases by comparing AI-generated plans with expert-planned and clinically placed implants [18]. AI showed no significant differences from human-based approaches in terms of angular deviation, coronal deviation, implant length, or implant diameter (all p > 0.05). Importantly, AI planning was considerably faster than expert planning and demonstrated greater consistency. These findings reinforce the results of the present study, in which the AI demonstrated good-to-excellent agreement with the expert consensus across implant dimensions, position, and angulation.

The clinical implications of the present study are important. The high level of agreement observed for implant dimensions suggests that AI may assist clinicians in selecting appropriate implant length and diameter while potentially reducing the variability associated with manual planning. The agreement observed for implant position and angulation further supports the potential utility of AI as an initial-planning aid. Such an approach may allow clinicians to generate a reproducible treatment proposal efficiently, which can subsequently be reviewed and modified according to individual anatomical, prosthetic, and clinical considerations of the patient. Importantly, AI should be regarded as a decision-support tool rather than a replacement for clinical expertise, particularly in anatomically complex cases.

Nevertheless, the lower exact agreement for implant position, and especially angulation, highlights an important clinical limitation. Implant angulation is influenced by prosthetic emergence, available bone, anatomical limitations, and clinician preference; therefore, small differences between AI and expert planning may not necessarily represent clinically meaningful differences. The absence of significant proportional bias in the Bland-Altman analyses is reassuring, but the relatively wide limits of agreement for angulation indicate that AI recommendations should not be regarded as automatically interchangeable with expert judgment in clinical practice. Current evidence similarly emphasizes that AI-based implant planning remains insufficiently validated for autonomous clinical use.

This study has several limitations. First, its retrospective, single-center design and relatively small sample of 50 edentulous sites may limit the generalizability of the findings, and the dataset may not fully represent the range of anatomical variations encountered in routine clinical practice. Second, only one posterior edentulous site per patient was evaluated; therefore, the findings may not be directly generalizable to anterior sites, multiple-unit edentulous spaces, severely resorbed ridges, immediate implant placement, or complex augmentation cases. Third, only CBCT datasets with adequate image quality and complete visualization of the relevant anatomical structures were included. Although necessary for consistent assessment, this criterion may have introduced selection bias by favoring relatively favorable datasets, potentially limiting applicability to more challenging clinical cases.

Fourth, the study assessed agreement with an expert consensus plan rather than the actual surgically placed implant. Thus, agreement with expert planning indicates similarity to the reference planning approach but does not establish surgical accuracy, clinical effectiveness, or improved patient outcomes. Fifth, the findings relate to the specific AI platform evaluated and may not be generalizable to other AI systems; furthermore, software updates or algorithmic modifications may affect reproducibility over time. Information regarding the underlying causes of tooth loss was also not consistently available in the retrospective records, limiting assessment of the representativeness of the sample according to the etiology of edentulism. Finally, planning time, clinician confidence, postoperative implant deviation, and long-term implant outcomes were not evaluated, and therefore potential effects of AI-assisted planning on workflow efficiency, clinician decision-making, surgical accuracy, and clinical outcomes remain to be established.

## Conclusions

This study demonstrated good-to-excellent agreement between AI-generated and expert consensus implant planning across implant length, diameter, position, and angulation. Most AI-generated recommendations fell within the predefined tolerance limits relative to the expert consensus plan. These findings support the potential use of AI as a planning adjunct for standardization and consistency of CBCT-based treatment proposals. However, agreement with expert consensus does not establish clinical or surgical accuracy. Prospective studies comparing AI-generated plans with actual postoperative implant positions are required to establish the clinical accuracy and applicability of AI-based implant planning.
